# Determinants of Noninvasive Ventilation Success or Failure in Morbidly Obese Patients in Acute Respiratory Failure

**DOI:** 10.1371/journal.pone.0097563

**Published:** 2014-05-12

**Authors:** Malcolm Lemyze, Pauline Taufour, Alain Duhamel, Johanna Temime, Olivier Nigeon, Nicolas Vangrunderbeeck, Stéphanie Barrailler, Gaëlle Gasan, Florent Pepy, Didier Thevenin, Jihad Mallat

**Affiliations:** 1 Department of Respiratory and Critical Care Medicine, Schaffner Hospital, Lens, France; 2 Department of Biostatistics, Lille University Hospital, CHRU Lille, France; 3 Respiratory Step Down Unit, Schaffner Hospital, Lens, France; D'or Institute of Research and Education, Brazil

## Abstract

**Purpose:**

Acute respiratory failure (ARF) is a common life-threatening complication in morbidly obese patients with obesity hypoventilation syndrome (OHS). We aimed to identify the determinants of noninvasive ventilation (NIV) success or failure for this indication.

**Methods:**

We prospectively included 76 consecutive patients with BMI>40 kg/m^2^ diagnosed with OHS and treated by NIV for ARF in a 15-bed ICU of a tertiary hospital.

**Results:**

NIV failed to reverse ARF in only 13 patients. Factors associated with NIV failure included pneumonia (n = 12/13, 92% vs n = 9/63, 14%; p<0.0001), high SOFA (10 vs 5; p<0.0001) and SAPS2 score (63 vs 39; p<0.0001) at admission. These patients often experienced poor outcome despite early resort to endotracheal intubation (in-hospital mortality, 92.3% vs 17.5%; p<0.001). The only factor significantly associated with successful response to NIV was idiopathic decompensation of OHS (n = 30, 48% vs n = 0, 0%; p = 0.001). In the NIV success group (n = 63), 33 patients (53%) experienced a delayed response to NIV (with persistent hypercapnic acidosis during the first 6 hours).

**Conclusions:**

Multiple organ failure and pneumonia were the main factors associated with NIV failure and death in morbidly obese patients in hypoxemic ARF. On the opposite, NIV was constantly successful and could be safely pushed further in case of severe hypercapnic acute respiratory decompensation of OHS.

## Introduction

Obesity has become a widespread disease worldwide, but the new alarming phenomenon is the emergence of an increasing number of individuals with extreme obesity [1]. As the body mass index (BMI) increases, so does the incidence of the multi-organ dysfunctions associated with obesity, especially the incidence of obesity hypoventilation syndrome (OHS) [2]. This syndrome refers to the association between obesity (BMI>30 kg.m^−2^) and daytime hypercapnia (PaCO_2_≥45 mmHg) after other respiratory or neuromuscular causes for such an awake hypoventilation have been excluded [3], [4]. The reported prevalence of OHS reaches 10 to 20% in the obese population while more than 50% of hospitalized patients with a BMI greater than 50 kg.m^−2^ meet the diagnostic criteria for OHS [2]. Considering obesity as a chronic multisystemic disease, some authors have recently used the term « malignant obesity hypoventilation syndrome » to refer to morbidly obese patients (BMI>40 kg/m^2^) with chronic alveolar hypoventilation, who exhibit a wide range of multi-organ dysfunctions, including obstructive sleep apnea syndrome, diabetes mellitus, metabolic syndrome, systemic hypertension, left ventricular hypertrophy, pulmonary hypertension, vitamin D deficiency [5], [6]. At this advanced stage, acute respiratory failure (ARF) is a common but nevertheless life-threatening event in the history of the disease. Although no randomized clinical trial has been conducted to test the effectiveness of noninvasive ventilation (NIV) for this indication, NIV is commonly delivered to these patients in the clinical setting and is considered standard of care [4], [7]–[10]. Most of the published studies in this field have focused on idiopathic exacerbations of OHS, systematically excluding patients with OHS admitted because of pneumonia, acute heart failure, pulmonary embolism, or any major cause of ARF [7]–[10]. We aimed to identify the determinants of NIV success or failure in morbidly obese patients with severe acute respiratory decompensation of OHS, whatever the cause of ARF.

## Materials and Methods

### Ethics Statement

This prospective observational study was conducted at the department of Emergency and Critical Care Medicine of the Schaffner Hospital during a two-year period, to evaluate all consecutive morbidly obese patients treated by NIV for ARF. The Schaffner Hospital ethics committee approved the study (approval number 07.02.11-1) and signed informed consent was obtained from all the patients or next of kin.

### Inclusion/Exclusion Criteria

Morbid obesity was defined according to the World Health Organization criteria by a body mass index (BMI) above 40 kg.m^−2^ [11]. Obesity hypoventilation syndrome was defined by the association between obesity and chronic alveolar hypoventilation resulting in daytime hypercapnia (PaCO_2_>45 mmHg) or elevated serum bicarbonate (>27 mmol/L), after the main causes of alveolar hypoventilation – such as severe obstructive or restrictive pulmonary diseases, neuromuscular diseases, or central hypoventilation – have been ruled out [4], [12].

As recommended, clinical criteria defining ARF included tachypnoea (>24 breaths per min), signs of increased work of breathing, accessory muscle use, and abdominal paradoxical motion [12]. According to arterial blood gas analysis at admission, three main profiles could be identified: pH<7.35, PaCO_2_>45 mm Hg, and PaO_2_/FiO_2_ ratio >200 defining hypercapnic ARF; pH>7.35, PaCO_2_<45 mm Hg, and PaO_2_/FiO_2_ ratio <200 defining hypoxemic ARF; pH<7.35, PaCO_2_>45 mm Hg, and PaO_2_/FiO_2_ ratio <200 defining mixed ARF. Patients could meet either clinical or blood gas criteria to be eligible to avoid delay in the application of assisted ventilation [13]. Causes for ARF were defined using clinical and imaging criteria according to recommendations respectively for the diagnosis and management of idiopathic exacerbation of OHS [4], pneumonia [14], acute heart failure [15], pulmonary embolism [16], and extrapulmonary sepsis [17].

The exclusion criteria included the absolute contraindications for NIV, i.e. respiratory or cardiac arrest and inability to fit a mask [13], another cause of chronic respiratory failure apart from obesity, BMI <40 kg.m^−2^, and a tracheotomy or endotracheal intubation performed before admission. Disorders of consciousness were not considered criteria for exclusion when the primary physician judged that they were exclusively related to hypercarbic encephalopathy.

### NIV Technique

NIV was started in the emergency room and was carried on in the ICU. According to our local protocol, the same turbine-driven portable ventilator (BiPAP Vision, Philips Respironics, Murrysville, PA) and oronasal mask (PerformaTrak, Philips Respironics, Murrysville, PA) were used in all patients [18]. The patients were gently placed in the sitting position and received reassuring explanations about the technique. The BiPAP Vision was equipped with its specific single branch circuit including an intentional leak, which was calibrated before each use and placed the closest as possible to the mask to prevent rebreathing phenomenon. Bilevel positive pressure targeted mode was delivered with the BiPAP Vision. The settings were adjusted at the discretion of the attending clinician. Active humidification (MR 850, Fischer & Paykel Healthcare, Auckland, New Zealand) was incorporated among the inspiratory circuit of the ventilator. NIV was continuously applied until a significant clinical improvement of the patient occurred. If effective, gradual reduction of the duration of NIV could be then considered by the attending physician.

### Evaluation Criteria

Anthropometric data, smoking history (pack years), severity of illness on admission assessed by the Sequential Organ Failure Assessment score [19] and by the Simplified Acute Physiologic Score II [20], and duration of NIV were recorded. Severity of respiratory disease was evaluated using Medical Research Council dyspnea score [21] 2 wk before admission by patient's or family's recall. Preadmission health status was assessed by Knaus index [22] 2 wk before admission and by Charlson's comorbidity score [23]. Encephalopathy score according to Kelly and Matthay scale [24], respiratory rate, heart rate, arterial blood pressure, arterial blood gases were measured before initiation of NIV, and after 1 to 2 hrs, 4 to 6 hrs, 12 hrs, 24 hrs, 48 hrs.

### Criteria Defining NIV Success and Failure

«Initial success» of NIV was defined according to objective and subjective criteria that reflect the patient's improvement in the first 48 hours following NIV initiation. The objective criteria included a decrease of ≥20% in respiratory rate compared with spontaneous breathing, an improvement in arterial blood gases with pH>7.35, a decrease in PaCO_2_ of ≥15% compared with spontaneous breathing while maintaining a SaO_2_ (with or without oxygen) ≥90%. The subjective criteria included improvement of the patient regarding both dyspnea and comfort [25].

« Delayed response to NIV » was defined as a significant clinical improvement of the patient leading to his/her recovery from ARF in the first 48 hrs, despite persistent respiratory acidosis after the first trial of facial mask-delivered NIV. Persistent respiratory acidosis referred to the inability to obtain a clinically significant decrease in PaCO_2_ of ≥15% (compared with the initial PaCO_2_ value under spontaneous breathing) or increase in pH>7.30 after 2 h of facial mask-delivered NIV.

« Early NIV failure » (<48 h) was defined according to major and minor criteria already used in the literature [26]. Major criteria included respiratory arrest, respiratory pauses or bradycardia (<50 bpm) with loss of consciousness, hypotension with systolic arterial blood pressure below 70 mm Hg, and refractory hypoxemia with inability to maintain a SaO_2_>90% despite high FiO_2_>60%. Minor criteria included tachypnea over 35 bpm or increase in the respiratory rate compared to its value at admission, pH<7.30 and decreased compared to its initial value, increase in the encephalopathy score compared to its initial value (according to Kelly et Matthay scale). Early NIV failure was considered when one major criteria was present, or when two minor criteria persisted after 6 h of NIV. When respiratory rate, encephalopathy score, PaCO_2_, or pH did not immediately improve with face mask-delivered NIV, the patient was correctly repositioned in the sitting position, and the interface could be changed to a total face mask [18]. If one or more minor criteria appeared after NIV had been resumed, NIV could be tried again. When NIV failed, escalation to endotracheal intubation was proceeded unless the patient had received a « do-not intubate » order. In that case, palliative care was initiated. According to standard of care and ethical practice of our hospital, patients received a do-not-intubate order when their physical disability and their underlying debilitating conditions made them poor candidates for intubation [18].

« Late failure » (>48 h) was defined as a sudden or gradual deterioration in arterial blood gases (pH<7.34 with an increase in PaCO_2_>15–20% compared to the previous value) with worsening of dyspnea, after an initial phase of partial recovery, even though NIV was pursued at least 6 h a day [25].

### Statistical Analysis

Data are presented as median (interquartile range, 25–75). Proportions were used as descriptive statistics for categorical variables. Data were not normally distributed according to the Shapiro-Wilks test and comparisons between independent groups were analyzed using the Mann-Whitney U test. The Friedman test was used to study the effect of NIV on PaCO_2_, pH, and PaO_2_/FiO_2_ over the time. The Bonferroni method was used to adjust for multiple comparisons. Analysis of the discrete data was performed by Fisher exact test. Statistical analyses were performed using SPSS (SPSS for windows release 17.0, Chicago, IL). A p value of less than 0.05 was considered statistically significant for single comparisons. All reported p values are two sided.

## Results

**Figure 1:**
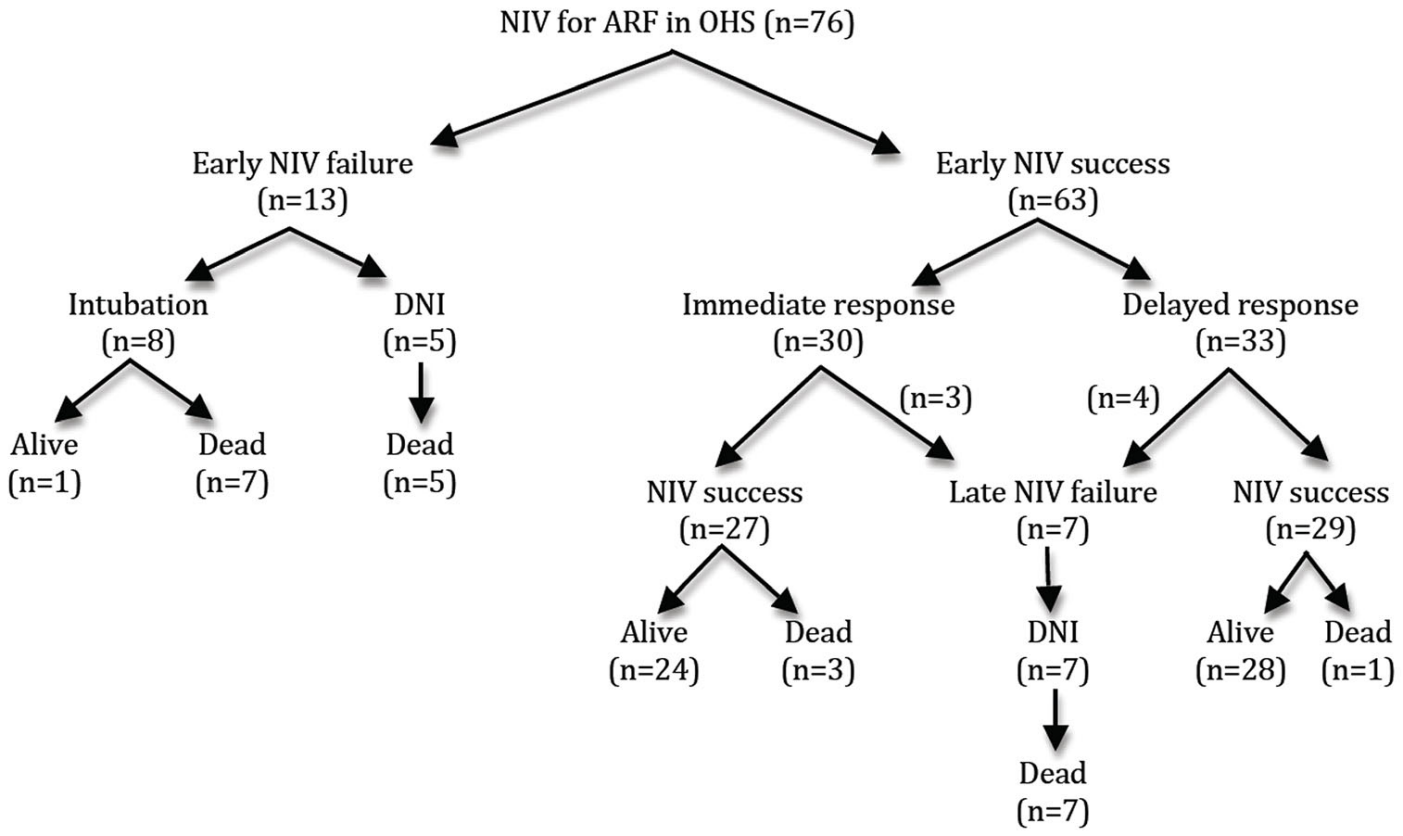
Flow chart showing patients' outcome according to early (within the first 48 hrs) or late (after the 48^th^ hr) NIV success or failure. NIV  =  noninvasive ventilation; ARF  =  acute respiratory failure; OHS  =  obesity hypoventilation syndrome; DNI  =  do-not-intubate.

We have studied 76 consecutive morbidly obese patients admitted to the ICU or to the step-down respiratory unit for ARF requiring NIV. Their main characteristics on admission are displayed in Table 1. In hospital mortality rate was 30%. NIV failed to reverse ARF in only 13 patients (17%). As shown in Figure 1, a significantly larger number of patients died during their hospital stay in the « early NIV failure » group compared to the « early NIV success » group (n = 12/13 vs. n = 11/63; p<0.0001). Patients of the « early NIV failure » group were more likely to be male with pneumonia, hypoxemic ARF, lower PaCO_2_ and HCO_3_ level, and higher severity scores at admission. Factors associated with successful response to NIV were high PaCO_2_ and HCO_3_ level at admission and idiopathic hypercapnic decompensation of OHS (Table 1). There was no difference between groups regarding functional status, comorbidities, and severity of respiratory disease before admission. The same number of patients was given a do-not-intubate order in the two groups (Table 1). Inspiratory positive airway pressure (IPAP, 18 [16]–[18] vs 18 [15]–[18] cm H_2_O; p = 0.99) and expiratory positive airway pressure (EPAP, 8 [6]–[10] vs. 8 [6]–[9] cm H_2_O; p = 0.75) were similar in the 2 groups. Respiratory depressant drugs were identified as a cofactor promoting ARF in 19 patients (n = 2/13, 15% vs. n = 17/63, 27%; p = 0.72). Hospitalization time was similar whatever the response to NIV (20 [3–43] vs 14 days [11]–[20]; p = 0.66), but patients for whom NIV failed had a longer stay in ICU compared to NIV success group (16 [1]–[22] vs 3 days [0–6]; p = 0.01).

**Table 1:** Patients characteristics at admission.

Parameters	Overall population (n = 76)	Early NIV success (n = 63)	Early NIV failure (n = 13)	p
Age (yrs)	63 [58–77]	63 [58–77]	66 [57–77]	NS
Male, n (%)	23 (31%)	18 (29%)	8 (61%)	0.05
Weight (kg)	134.5 [120–159]	135 [120–160]	134 [115–146]	NS
BMI (kg/m^2^)	49.6 [45–57]	50 [45–57]	49 [43–52]	NS
Albumine (g/L)	27.5 [23]–[31]	28 [23]–[31]	26 [23]–[32]	NS
MRC dyspnea score	5 [4]–[5]	5 [4]–[5]	5 [4]–[5]	NS
Knaus index C, n (%)	23 (30%)	16 (25%)	7 (54%)	0.05
Knaus index D, n (%)	41 (54%)	36 (57%)	5 (38%)	NS
Charlson comorbidity score	4 [3]–[5]	4 [3]–[5]	4 [3.5–5]	NS
Diabetes mellitus, n (%)	62 (82%)	51 (81%)	11 (85%)	NS
Hypertension, n (%)	69 (91%)	57 (90%)	12 (92%)	NS
OSAS, n (%)	63 (83%)	53 (84%)	10 (77%)	NS
Indication for NIV, n (%)				
Hypercapnic ARF	38 (52%)	35 (57%)	3 (23%)	0.03
Hypoxemic ARF	10 (14%)	6 (10%)	4 (31%)	0.04
Mixed ARF	27 (34%)	21 (34%)	6 (46%)	NS
Causes for ARF, n (%)				
Idiopathic	30 (39.5%)	30 (48%)	0 (0%)	0.001
Pneumonia	21 (28%)	9 (14%)	12 (92%)	<0.0001
Acute heart failure	10 (13%)	10 (16%)	0 (0%)	NS
Sepsis	5 (7%)	4 (6%)	1 (8%)	NS
Pulmonary embolism	1 (1.3%)	1 (2%)	0 (0%)	NS
Other	9 (12%)	9 (14%)	0 (0%)	NS
DNI status, n (%)	44 (58%)	39 (62%)	5 (38%)	NS
SAPS 2	41 [34–53]	39 [33–47]	63 [53–72]	<0.0001
SOFA score	5 [4]–[7]	5 [4]–[6]	10 [7–11.5]	<0.0001
Respiratory rate (bpm)	25 [21]–[30]	25 [20]–[30]	25 [22]–[36]	NS
Encephalopathy score	4 [3]–[4]	4 [3]–[4]	3 [3]–[4]	NS
SABP (mm Hg)	132 [116–156]	135 [117–156]	119 [102–152]	NS
Arterial blood gases				
pH	7.27 [7.21–7.33]	7.26 [7.20–7.33]	7.28 [7.27–7.37]	NS
PaCO_2_ (mm Hg)	72 [61–86]	74 [62–88]	61 [43–73]	0.01
PaO_2_ (mm Hg)	63 [52–85]	66 [53–85]	59 [48–76]	NS
PaO_2_/FiO_2_	209 [157–260]	211 [159–262]	167 [137–238]	NS
HCO_3_^−^ (mmol/L)	31 [26]–[39]	32 [27]–[40]	28 [24]–[30]	0.02

Abbreviations: BMI, body mass index; NIV, non-invasive ventilation; ARF, acute respiratory failure; MRC, Medical Research Council dyspnea score; OSAS, obstructive sleep apnea syndrome; DNI status, do-not-intubate status; SAPS 2, simplified acute physiologic score 2; SOFA score, sequential organ failure assessment score; SABP, systolic arterial blood pressure.

**Figure 2:**
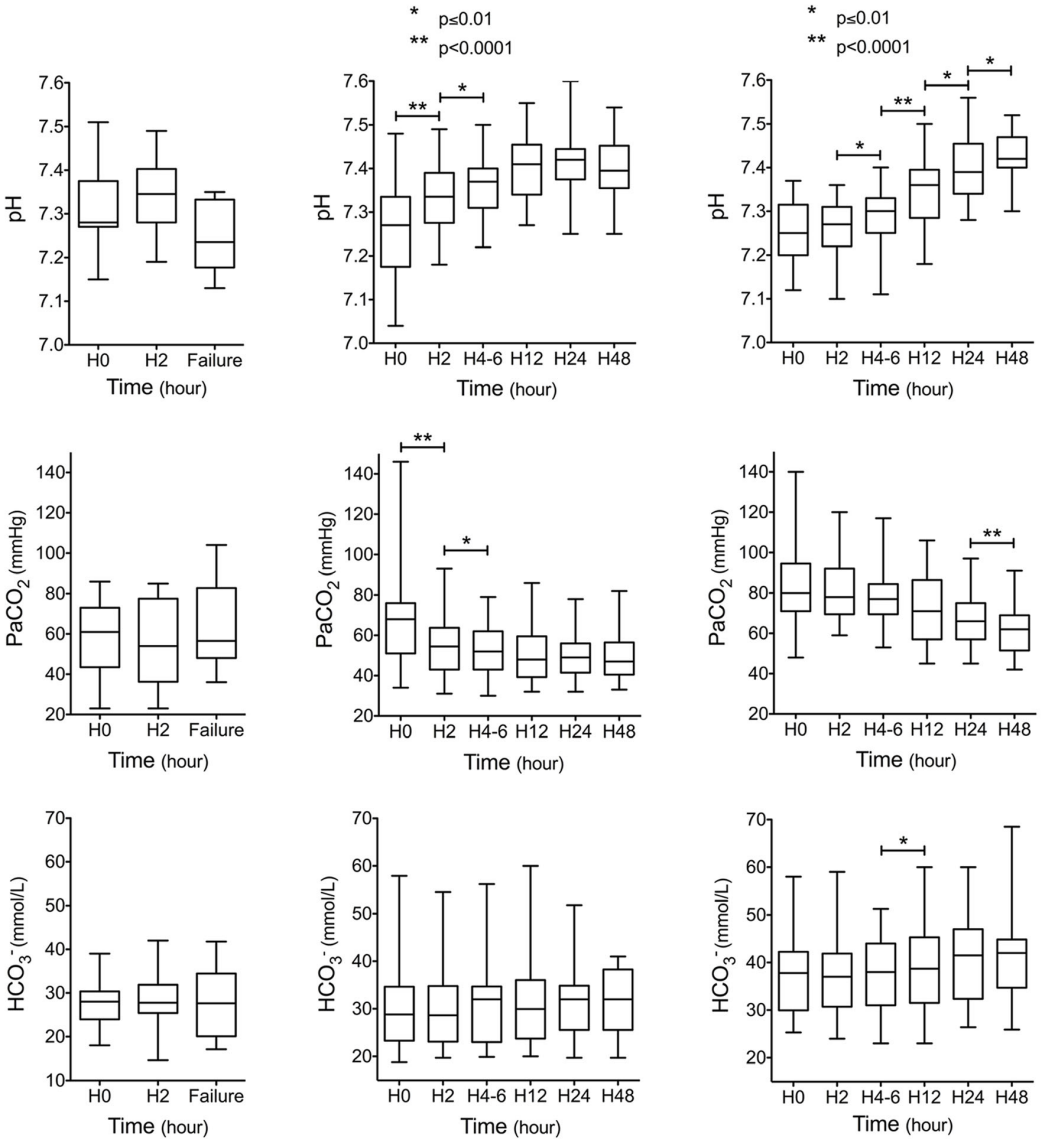
Arterial blood gases at the time of noninvasive ventilation (NIV) initiation (H0), after 2 hrs of NIV (H2), and at the time of NIV failure (failure) in patients of the early NIV failure group (n = 13, left column). By comparison, arterial blood gases evolution during the first 48(n = 30, central column) and in patients with a delayed response to NIV (n = 33, right column).

More than half of the patients of the early NIV success group (n = 33/63) exhibited a “delayed response to NIV” with persistent respiratory acidosis after the first hours of NIV. Arterial blood gases response to NIV over the first 48 hours is shown in Figure 2 according to patients group (« early NIV failure » vs « immediate response to NIV » vs « delayed response to NIV »). Among the early NIV success group, patients demonstrating a delayed response to NIV were more likely to be treated by diuretics in the ICU (n = 16/33, 48.5% vs n = 6/30, 20%; p = 0.02) or to have respiratory depressant drugs as part of their medication regimen before admission (n = 15/33, 45.5% vs n = 2/30, 7%; p = 0.001). On the contrary, EPAP (8 [6]–[8] vs 8 [6]–[10] cmH_2_O; p = 0.378) and IPAP (18 [16]–[20] vs 17 [14]–[18] cmH_2_O; p = 0.14) were similar in patients with a delayed response to NIV (n = 33) as compared to immediate responders to NIV (n = 30).

## Discussion

The main results of this study can be summarized as follows:

1. In morbidly obese patients with decompensated OHS, NIV rarely failed in reversing ARF.
2. Morbidly obese patients who exhibited early NIV failure had a high severity score and a low HCO_3_ level at admission, and they were likely to have hypoxemic ARF caused by pneumonia.
3. Factors associated with a successful response to NIV included high PaCO_2_ at admission and a diagnosis of idiopathic hypercapnic decompensation of OHS.
4. More than half of the hypercapnic patients with decompensated OHS exhibited a delayed response to NIV.

Extreme obesity is a major cause of chronic respiratory failure. Unfortunately, it is often underrecognized and misdiagnosed, even though morbidly obese patients are admitted to the ICU for ARF [3], [6], [27]. By recently describing malignant OHS, Marik et al have shown that more than 75% of these patients were erroneously diagnosed and treated for COPD or asthma exacerbation [6]. Malignant obesity hypoventilation syndrome deserves more attention as it carries a non-negligible risk of in-hospital mortality [6], [28]–[30]. In the present study, the reported population of morbidly obese patients is very close to the one described by Marik et al. Almost all our patients had diabetes mellitus, obstructive sleep apnea syndrome, hypertension, and 30% of them died in hospital despite early initiation of mechanical ventilation. This dramatic outcome may be explained both by the high severity scores at admission and the characteristics of the studied population. Eighty percent of our patients were already bedridden or overdependent according to Knaus classification before admission to hospital. Most of them exhibited numerous serious illnesses as demonstrated by a very high Charlson comorbidity index. Not surprisingly, given their very impaired functional status and multiple debilitating conditions, about 60% of those morbidly obese patients were given a « do-not-intubate » order. The DNI status of these frail patients may partly explain the high mortality associated with NIV failure in the present study. However, considering early NIV failure group (n = 13), 8 patients were intubated, and despite no limitation decision, only one survived hospital discharge (Figure 1). Patients in whom NIV initially succeeded in reversing ARF in the critical care setting may nevertheless experience a second episode of ARF once they are transferred to the ward. In this study, 8 DNI patients exhibited this scenario and all of them eventually died. The disastrous outcome of a second episode of ARF (“late failure”) has already been described for COPD patients treated by NIV [25]. Despite proper and loyal information regarding the need for further NIV, half of these frail morbidly obese patients refused continuation of NIV. From an ethical point of view, NIV must be considered as an artificial life-supporting modality that cannot be imposed by force. In the other four, late NIV failure may result from a smaller nurse-to-patient ratio in the ward and may reflect end-stage chronic respiratory failure.

NIV is usually used in an attempt to avoid intubation and the numerous complications occurring during the course of prolonged invasive mechanical ventilation, such as ventilator-associated pneumonia, delirium, muscle weakness, and eventually death [13]. On the other hand, NIV is still a questionable issue for patients in severe hypoxemic ARF. In our study, patients of the early NIV failure group were most likely to have pneumonia, and multiple organ failure at admission. Some may argue that prolonging NIV in non-responders may dangerously delay intubation and thus increase the risk of death, especially in patients with ARDS or hypoxemic ARF due to pneumonia [31], post-extubation ARF [32], or COPD patients developing « late NIV failure » [25]. It should be stressed that, in our protocol, refractory hypoxemia and hemodynamic instability were part of the major criteria defining early NIV failure and indicating the need for urgent tracheal intubation. As a result, none of the hypoxemic patients of our study experienced a delayed implementation to intubation, when it was indicated. In the specific situation of massively obese patients with hypoxemic ARF, at best NIV avoids intubation, at worst it can be used to pre-oxygenate before intubation [33]. The deleterious effects of massive obesity on respiratory physiology – i.e. abnormal upper airway collapsibility [34], reduced lung volume and expiratory flow limitation promoted by supine position [35], increased work of breathing [36], ventilation-perfusion mismatch worsened by gravitational atelectasis [37] and extrinsic compression of the thorax by the abdominal compartment [38] – increase the risk of rapid arterial oxygen desaturation and jeopardize the process of weaning from mechanical ventilation, once intubation has been performed. Furthermore, intubated patients usually require sedation and confinement to bed, two situations that lead to lung derecruitment and thus may worsen hypoxemia in morbidly obese patients [38]. All these arguments may explain the high mortality of the few morbidly obese patients intubated in our study.

As already demonstrated [7], [8], arterial blood gases significantly improved with NIV over the first 48 hrs in morbidly obese patients with hypercapnic ARF (Figure 2). Nevertheless, considering arterial blood gases on a case-by-case basis, about half of the hypercapnic patients could have been considered initially as poor responders given their unimproved PaCO_2_ and pH after the first trial of NIV. According to the criteria chosen to define early NIV failure in this study, NIV was pursued, though. None of these patients was eventually intubated, and non-invasive treatment succeeded in reversing hypercapnic ARF in all of them. Our data suggest that both pH and PaCO_2_ may not accurately predict hypercapnic obese patients response to NIV during the first hours of therapy. As demonstrated here, a substantial number of morbidly obese patients in hypercapnic ARF exhibit a delayed response to NIV. Our results suggest that further NIV rather than immediate intubation could be safely considered in the ICU for morbidly obese patients still in severe hypercapnic ARF after the first hours of NIV. The decreased responsiveness in hypoxic and hypercapnic ventilatory drive that characterizes patients with OHS [39] may explain why they need more time than expected to correct respiratory acidosis with NIV. Another explanation might be given by the medications used that may promote or prolong alveolar hypoventilation in obese patients. Diuretic therapy is largely used in patients with decompensated OHS in an attempt to reverse anasarca and to unload the right ventricle especially in the setting of acute cor pulmonale. Raurich et al have demonstrated a more blunted CO_2_ response in patients with OHS and higher bicarbonate concentrations [40]. Diuretics by promoting metabolic alkalosis may worsen alveolar hypoventilation, prolonging the need for respiratory support.

There are several potential limitations that we have to acknowledge. First, given the prospective observational study design, selection bias cannot be strictly ruled out. Secondly, the results of such a single center study may not be reproducible in other centers with a different approach to NIV. Aware of the undeniable beneficial effects of NIV in massively obese patients in hypercapnic ARF, all the members of our team are doing everything in their power to ensure this subset of patients can tolerate NIV as long as possible. Reassuring explanations about the technique, patient's setup and confort, the choice of the interface, facial skin protection, and adapting the ventilator settings according to patient's need are priority issues in the daily practice of NIV in our department [18]. Of course, the results of such a study aiming at identifying factors that influence NIV outcome highly depend on how NIV success and failure are defined. Therefore, we used previously validated definitions to assess patients' response to NIV [25], [26]. Finally, from a statistical point of view, the small number of events (only 13 NIV failure events out of 76 morbidly obese patients included) prevents any multivariate analysis of our results [41]. Assuming that 4 variables are associated with NIV failure in univariate analysis – as in the present study – and the ratio of events per variable in multiple logistic regression analysis should be at least ten [41], 234 morbidly obese patients should be included to perform such a statistical analysis. Given that it is the first study investigating the factors associated with NIV success or failure in a field of limited knowledge, the present study may be considered more as a pilot study, and can serve as a support for calculating the sample size requested for future studies. Large multicenter prospective trials are needed to confirm our results in a broader population of morbidly obese patients in ARF.

Severe pneumonia and multiple organ failure often caused early NIV failure in massively obese patients in hypoxemic ARF. On the contrary, NIV was constantly successful in reversing idiopathic hypercapnic ARF in this subset of patients. Considering that more than half of morbidly obese patients in hypercapnic ARF exhibit a delayed response to NIV, NIV should be pushed further for this indication.
